# New observations on rare and threatened saproxylic hoverflies in North-Western Italy with ecological notes

**DOI:** 10.3897/BDJ.12.e127968

**Published:** 2024-09-27

**Authors:** Umberto Maritano, Lorenzo Bianco, Samuele Torta

**Affiliations:** 1 Department of Life Science and Systems Biology, University of Turin, Turin, Italy Department of Life Science and Systems Biology, University of Turin Turin Italy

**Keywords:** Syrphidae, Piedmont, *Hederahelix*, pollinators, Sap-run, endangered species

## Abstract

**Background:**

The knowledge on the distribution of saproxylic hoverflies in the Piedmont region has always been incomplete, despite the large wooded areas present in the territory. We know of a few scattered specimens collected in the 19^th^ century, often with a generic or incomplete locality on the label such as “surrounding Turin” or “Alps”. The recent publication of the first European Red List on hoverflies highlights some threatened species including some saproxylics. To fill this knowledge gap we report here unpublished data and establish new ecological note on threatened or nationally scarce saproxylic species actually present in the region.

**New information:**

In this study, we present new findings concerning rare saproxylic hoverflies. These include the rediscovery of *Hammerschmidtiaferruginea* (Fallen), 1817, marking its first recorded occurrence in the Italian fauna since the middle of 19^th^ century. Additionally, we report novel observations of the "Chimera fly" *Dorosdestillatorius* Mik, 1885, along with further observations on endangered or vulnerable species such as *Callicera* spp. and *Brachypalpuschrysites* Egger 1859. Moreover, we document the rediscovery of *Criorhinapachymera* Egger, 1858, within the Piedmont region, and the first record of *Brachyopagrunewaldensis* Kassebeer, 2000, within Italy. Comprehensive data detailing these observations are provided herein.

## Introduction

In this paper, we summarise the knowledge on the 6 species threatened with extinction actually present in Piedmont, with the addition of two rare species of national relevance. We do not include here *Brachyopamaculipennis* Thompson, 1980 because we don’t have new data and it is possibly extinct. We provide full data including behaviour and ecological notes in order to better understand the distribution and ecology of these species.

## Materials and methods

We present data collected through observations made in the field by the authors in the last 5 years of research in the Piedmont region on exceptionally rare, threatened or new saproxylic hoverflies species for this territory. We also show previous published and unpublished data for the 8 selected species to give a comprehensive overview. Label data are provided in chronological order for every species. Photos were taken in the field by the authors. Additional data on habitat type, behaviour and collection conditions is reported when available.

## Taxon treatments

### Brachyopa grunewaldensis

Kassebeer, 2000

AF6373E1-AB2E-56D2-9853-ABB973304C3B

#### Materials

- **Type status:** Other material. **Occurrence:** individualCount: 1; sex: female; lifeStage: adult; behavior: roosting; occurrenceID: 86DDAF45-B466-5BDD-B538-4F552096A609; **Location:** country: Italy; stateProvince: Piedmont; municipality: La Cassa (TO); verbatimElevation: 393 m; verbatimLatitude: 45°11'59'' N; verbatimLongitude: 7°31'36'' E; verbatimCoordinateSystem: WGS84; decimalLatitude: 45.1997222; decimalLongitude: 7.5266666; geodeticDatum: WGS84; **Identification:** identifiedBy: Umberto Maritano; identificationReferences: van Steenis et al., 2020; **Event:** samplingProtocol: ad hoc observation; eventDate: 2024-04-11; eventTime: 17:30:00Z; habitat: Oak-hornbeam forest; eventRemarks: on a sap-run of a oak; **Record Level:** type: PhysicalObject; collectionID: Umberto Maritano collection

#### Notes

**Figure 1.**
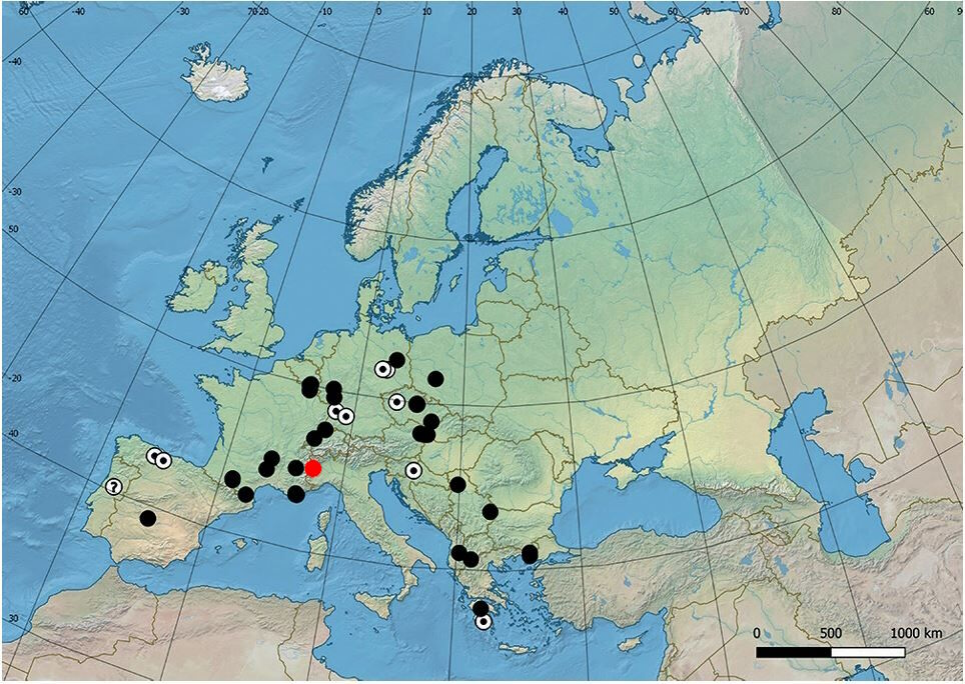
Distribution map of Brachyopagrunewaldensis white <1950, white with black point ≥1950 <2000, black ≥2000, ? = uncertain record. Map modified from Steenis et al. (2020) with a red dot for new data.

**Figure 2.**
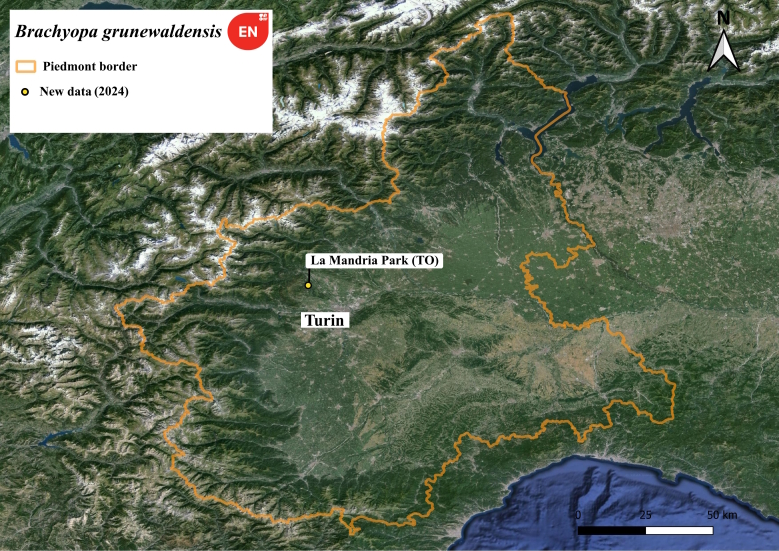
Distribution map of *Brachyopagrunewaldensis* in Piedmont region.

This is an endangered species (IUCN 2021) and our specimen is the first report for the Italian fauna (Fig. 1). A single female was collected by one of the authors (UM) in La Mandria Natural Park (Fig. 2) on a sap-run on a large *Quercus* tree at 5:30 p.m. The observation is in accordance with Speight 2020 who reported late afternoon activity for this rare species.

### Brachypalpus chrysites

Egger, 1859

2E962828-5799-5100-B1B5-C37D82A01F2B

#### Materials

- **Type status:** Other material. **Occurrence:** individualCount: 1; sex: female; lifeStage: adult; occurrenceID: 3718361D-DBF9-52DA-ACDA-DCB46F95426D; **Location:** country: Italy; stateProvince: Piedmont; municipality: Valdieri (CN); locality: Piana della casa di caccia; verbatimElevation: 1757 m; verbatimLatitude: 44°12'01'' N; verbatimLongitude: 7°14'20'' E; verbatimCoordinateSystem: WGS84; decimalLatitude: 44.2002777; decimalLongitude: 7.2388888; geodeticDatum: WGS84; **Identification:** identifiedBy: Umberto Maritano; **Event:** samplingProtocol: Malaise trap; eventDate: 2022-06-02/16; habitat: coniferous forest; **Record Level:** type: PhysicalObject; collectionID: Umberto Maritano collection

#### Notes

**Figure 3.**
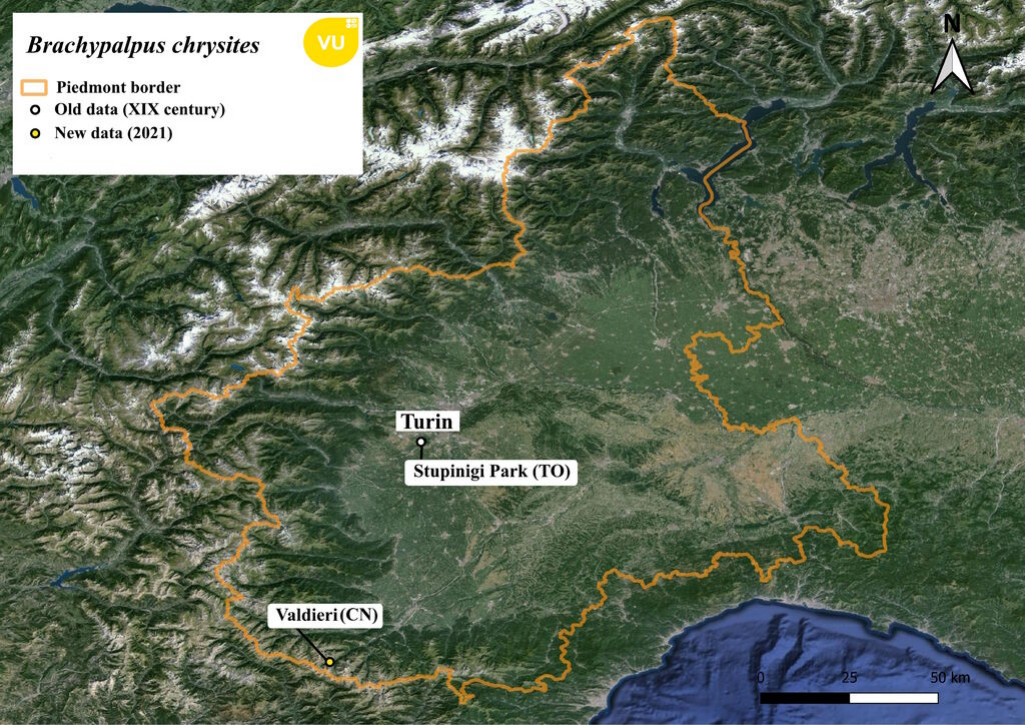
Distribution map of *Brachypalpuschrysites* in Piedmont region.

This species is vulnerable according to the IUCN European Red List of Hoverflies (Vujić et al. 2022). It was recorded in the 19^th^ century (Sommaggio 2007) on a coniferous plantation at low altitude in Stupinigi Park (Fig. 3), but a recent attempt made by the authors failed to find it in the same place. A specimen was collected by Malaise trap in a subalpine pastured grassland in Valasco Valley (CN) during the second year of a survey concerning the effect of grazing on hoverfly assemblages. Other observations that took place regularly in the same year at higher altitudes (2040 m a.s.l.) on a senescent coniferous plant have led to the observation of a single *Brachypalpusvalgus* specimen.

### Callicera aurata

(Rossi, 1790)

516D14BF-2387-5E5F-BB10-28F2CB41FC07

#### Materials

- **Type status:** Other material. **Occurrence:** individualCount: 1; sex: male; lifeStage: adult; behavior: foraging; occurrenceID: EB554C3C-E53F-5C50-8978-E2EFC1CE5397; **Location:** country: Italy; stateProvince: Piedmont; municipality: Bioglio (BI); locality: Rondolere; verbatimElevation: 1190 m; verbatimLatitude: 45°40'41'' N; verbatimLongitude: 8°02'42'' E; verbatimCoordinateSystem: WGS84; decimalLatitude: 45.6780555; decimalLongitude: 8.045; geodeticDatum: WGS84; **Identification:** identifiedBy: Umberto Maritano; **Event:** samplingProtocol: entomological net; eventDate: 2019-08-13; habitat: Fagus forest; **Record Level:** type: PhysicalObject; collectionID: Umberto Maritano Collection
- **Type status:** Other material. **Occurrence:** individualCount: 1; sex: female; lifeStage: adult; behavior: foraging; occurrenceID: 491DD818-EB6C-5D0B-9126-07CC8D530813; **Location:** country: Italy; stateProvince: Piedmont; municipality: Armeno (NO); verbatimElevation: 962 m; verbatimLatitude: 45°50'34'' N; verbatimLongitude: 8°28'22'' E; verbatimCoordinateSystem: WGS84; decimalLatitude: 45.8427777; decimalLongitude: 8.4727777; geodeticDatum: WGS84; **Identification:** identifiedBy: Umberto Maritano; **Event:** samplingProtocol: observation; eventDate: 2006-08-30; habitat: Deciduous mixed forest; eventRemarks: iNaturalist; **Record Level:** type: Stillimage
- **Type status:** Other material. **Occurrence:** individualCount: 1; sex: female; lifeStage: adult; behavior: foraging; occurrenceID: AED2843A-72B8-54B2-AB2A-E71E1FD56496; **Location:** country: Italy; stateProvince: Piedmont; municipality: Ormea (CN); verbatimElevation: 1792 m; verbatimLatitude: 44°08'42'' N; verbatimLongitude: 7°50'56'' E; verbatimCoordinateSystem: WGS84; decimalLatitude: 44.145; decimalLongitude: 7.8488888; geodeticDatum: WGS84; **Identification:** identifiedBy: Umberto Maritano; **Event:** samplingProtocol: observation; eventDate: 2020-08-15; habitat: Open grassland high altitude; eventRemarks: iNaturalist; **Record Level:** type: Stillimage
- **Type status:** Other material. **Occurrence:** individualCount: 1; sex: female; lifeStage: adult; behavior: foraging; occurrenceID: 2A1D9CBB-D2DA-508B-A155-7B1AE7323D41; **Location:** country: Italy; stateProvince: Piedmont; municipality: Oncino (CN); verbatimElevation: 1723 m; verbatimLatitude: 44°39'47'' N; verbatimLongitude: 7°09'40'' E; verbatimCoordinateSystem: WGS84; decimalLatitude: 44.6630555; decimalLongitude: 7.1611111; geodeticDatum: WGS84; **Identification:** identifiedBy: Umberto Maritano; **Event:** samplingProtocol: entomological net; eventDate: 2020-07-18; habitat: Open grassland high altitude; **Record Level:** type: PhysicalObject; collectionID: Umberto Maritano Collection
- **Type status:** Other material. **Occurrence:** individualCount: 1; sex: female; lifeStage: adult; behavior: foraging; occurrenceID: 1735F323-CC03-583C-9F03-158F3A01148C; **Location:** country: Italy; stateProvince: Piedmont; municipality: Frabosa Sottana (CN); verbatimElevation: 573 m; verbatimLatitude: 44°19'10'' N; verbatimLongitude: 7°48'13'' E; verbatimCoordinateSystem: WGS84; decimalLatitude: 44.3194444; decimalLongitude: 7.8036111; geodeticDatum: WGS84; **Identification:** identifiedBy: Lorenzo Bianco; **Event:** samplingProtocol: entomological net; eventDate: 2022-07-18; habitat: Deciduous mixed forest; **Record Level:** type: PhysicalObject; collectionID: Lorenzo Bianco Collection
- **Type status:** Other material. **Occurrence:** individualCount: 1; sex: female; lifeStage: adult; behavior: flying; occurrenceID: 451A1387-B4BF-528B-A7E1-D22C58FEC273; **Location:** country: Italy; stateProvince: Piedmont; municipality: Nucetto (CN); verbatimElevation: 447 m; verbatimLatitude: 44°20'50'' N; verbatimLongitude: 8°03'21'' E; verbatimCoordinateSystem: WGS84; decimalLatitude: 44.3472222; decimalLongitude: 8.0558333; geodeticDatum: WGS84; **Identification:** identifiedBy: Lorenzo Bianco; **Event:** samplingProtocol: entomological net; eventDate: 2022-08-03; habitat: Deciduous mixed forest; **Record Level:** type: PhysicalObject; collectionID: Umberto Maritano Collection

#### Notes

**Figure 4.**
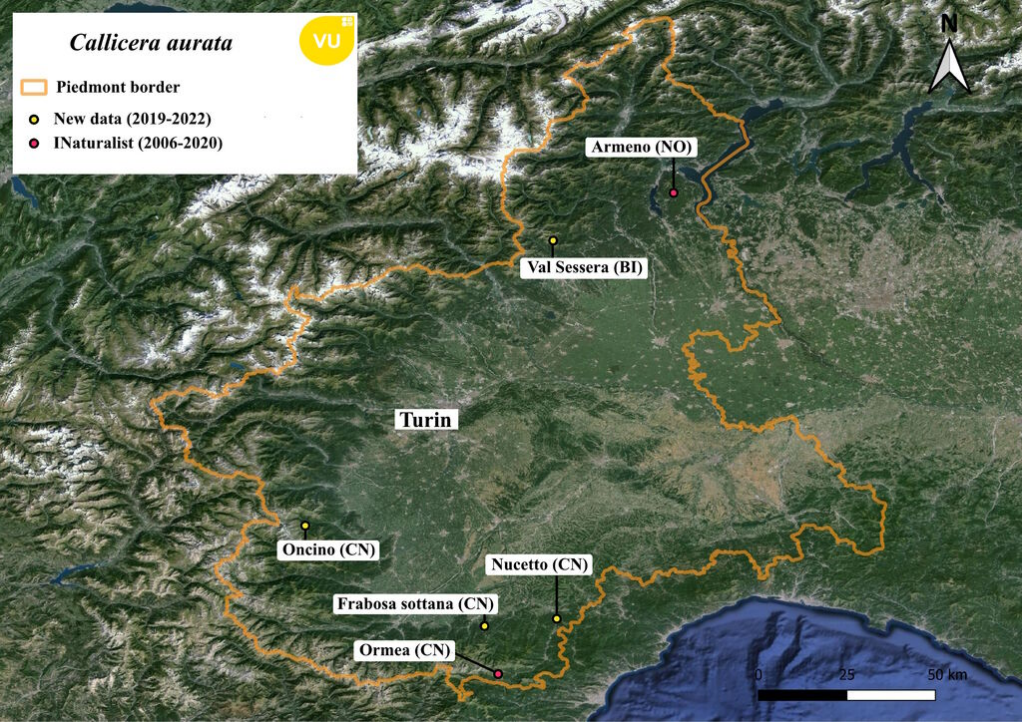
Distribution map of *Calliceraaurata* in Piedmont region.

This species is reported as vulnerable (IUCN 2021) and all the data known for the Piedmont region were unpublished (Fig. 4). Two observations come from the iNaturalist platform (checked and confirmed by the authors) and four from authors' (UM, LB) observations. All the specimens collected by the authors were observed during the hottest hours of the day, all on different flowers: the specimen from Sessera Valley was observed on violet *Knautia* sp. or *Scabiosa* sp., the specimen in Oncino was observed on *Hypericum* sp., the specimen in Frabosa Sottana was on white *Dipsacus* sp. and the one in Nucetto was near *Fragariavesca*. While for iNaturalist observation the specimen from Ormea was on *Scabiosa* sp. while the specimen from Armeno was on *Carduus* sp. or *Cirsium* sp.

### Callicera fagesii

Guerin-Meneville, 1844

D1FF153A-D1C6-5DDA-81A1-09D4AB2C1FF4

#### Materials

- **Type status:** Other material. **Occurrence:** individualCount: 1; lifeStage: adult; behavior: roosting on bark; occurrenceID: 575B16CF-9DB3-5DFB-922D-0A475BCF8B1B; **Location:** country: Italy; stateProvince: Piedmont; municipality: Venaria Reale (TO); locality: La Mandria Park; verbatimElevation: 307 m; verbatimLatitude: 45°09'03'' N; verbatimLongitude: 7°35'49'' E; verbatimCoordinateSystem: WGS84; decimalLatitude: 45.1508333; decimalLongitude: 7.5969444; geodeticDatum: WGS84; **Event:** samplingProtocol: ad hoc observation; eventDate: 2024-04-08; habitat: Oak-hornbeam forest; eventRemarks: basking on the bark of old Quercus at 3 m from ground for a few seconds; **Record Level:** type: Observation
- **Type status:** Other material. **Occurrence:** individualCount: 1; sex: female; lifeStage: adult; behavior: flying near *Crataegus* bloom; occurrenceID: CF47DDA4-C10C-5095-B078-A8C316E2FCC1; **Location:** country: Italy; stateProvince: Piedmont; municipality: San Gillio (TO); locality: Lago Bonino; verbatimElevation: 350 m; verbatimLatitude: 45°07'45'' N; verbatimLongitude: 7°30'43'' E; verbatimCoordinateSystem: WGS84; decimalLatitude: 45.1291666; decimalLongitude: 7.5119444; geodeticDatum: WGS84; **Identification:** identifiedBy: Umberto Maritano; **Event:** samplingProtocol: entomological net; eventDate: 2024-04-18; habitat: Alluvial forest with *Quercus*; eventRemarks: near *Crataegus* bloom; **Record Level:** type: PhysicalObject; collectionID: Umberto Maritano Collection

#### Notes

**Figure 5.**
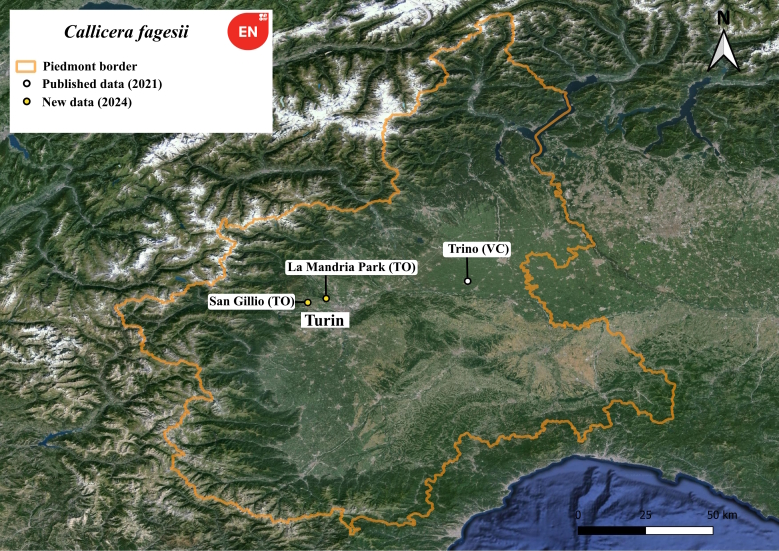
Distribution map of *Callicerafagesii* in Piedmont region.

This is an endangered species (Vujić et al. 2022) collected only recently for the Piedmont region (Maritano 2021). The female specimen was observed foraging on *Crataegus* flower on 29 April 2020 during the hottest hours of the day (See white dot in Fig. 5). At La Mandria Natural Park (Venaria Reale, TO) a specimen was observed basking on the bark of a large *Quercus* tree 3 metres above the ground during the afternoon around 4:30 pm, but we failed to collect it. Although not captured, we can say with certainty that it was a *Callicerafagesii* specimen as it is the only *Callicera* species present at that time of year in such Habitat type. This is confirmed by the last observation that occurred 10 days later in San Gillio Lake Natural Reserve with a specimen found near a blooming *Crataegus* at 12:30 am.

### Callicera spinolae

Rondani, 1844

8524B6E5-7EE5-58B9-8A8D-FB3CEE60C065

#### Materials

- **Type status:** Other material. **Occurrence:** individualCount: 1; sex: male; lifeStage: adult; preparations: pinned; occurrenceID: 497FA59D-ED1C-5123-A580-E31D0334C394; **Location:** country: Italy; stateProvince: Piedmont; locality: Moncalieri (TO); **Identification:** identifiedBy: Lorenzo Bianco; Umberto Maritano; **Event:** samplingProtocol: pinned specimen; eventDate: 1938-09; **Record Level:** type: Dry pinned specimen; collectionID: University of Turin DISAFA agricultural entomology collection, Grugliasco (TO)
- **Type status:** Other material. **Occurrence:** individualCount: 1; lifeStage: adult; behavior: foraging; occurrenceID: C85DD63B-0834-587E-8659-F531A0EAD862; **Location:** country: Italy; stateProvince: Piedmont; municipality: Bussoleno (TO); locality: Foresto hamlet; verbatimElevation: 513 m; verbatimLatitude: 45°08'34'' N; verbatimLongitude: 7°06'52'' E; verbatimCoordinateSystem: WGS84; decimalLatitude: 45.1427777; decimalLongitude: 7.1144444; geodeticDatum: WGS84; **Identification:** identifiedBy: Umberto Maritano; **Event:** samplingProtocol: ad hoc observation; eventDate: 2023-09-19; eventTime: 16:30; eventRemarks: foraging on *Hederahelix*; **Record Level:** type: Observed

#### Notes

**Figure 6.**
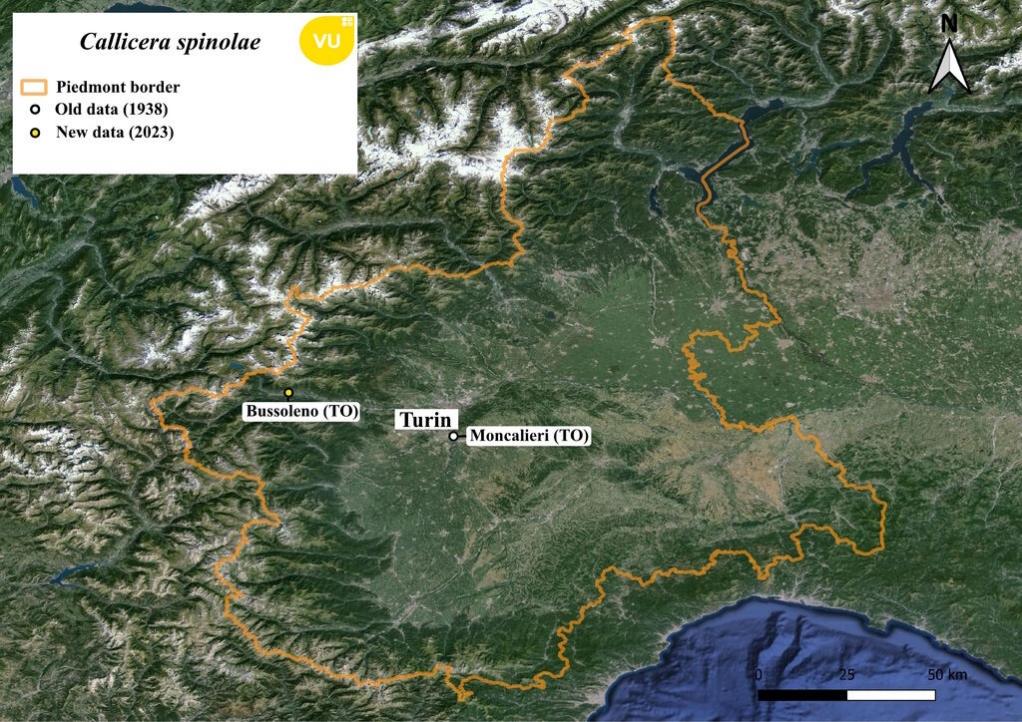
Distribution map of *Calliceraspinolae* in Piedmont region.

*Calliceraspinolae* is a vulnerable species (Vujić et al. 2022) and it is reported here as a new species for the Piedmont region fauna with an old unpublished specimen found with the label “Moncalieri IX 1938 Dr. F. Festa”, dry and pinned in the University of Turin DISAFA collection in Grugliasco (TO), and an observed specimen in Susa Valley (Fig. 6). In Susa Valley the specimen was observed at 5:00 pm on a large ivy bloom (the same reported below in *Dorosdestillatorius*) but we missed collecting it and despite other attempts to find it on subsequent days it never came back to the same plant. Although not captured, we can say with certainty that it was a *Calliceraspinolae* specimen as it is the only *Callicera* species present in this type of habitat.

### Criorhina pachymera

Egger, 1858

69160ABA-609F-5C3C-97D7-71F358139DE5

#### Materials

- **Type status:** Other material. **Occurrence:** individualCount: 1; sex: male; lifeStage: adult; reproductiveCondition: mating; behavior: mating on bark of *Populus*; occurrenceID: 8B0338D1-1713-5FEF-863F-0E1AD5EB4852; **Location:** country: Italy; municipality: Rocca de' Baldi (CN); locality: Crava-Morozzo Natural Reserve; verbatimElevation: 410 m; verbatimLatitude: 44°25'21'' N; verbatimLongitude: 7°43'26'' E; verbatimCoordinateSystem: WGS84; decimalLatitude: 44.4225; decimalLongitude: 7.7238888; geodeticDatum: WGS84; **Identification:** identifiedBy: Samuele Torta; **Event:** samplingProtocol: ad hoc observation; eventDate: 2024-04-25; **Record Level:** type: Stillimag
- **Type status:** Other material. **Occurrence:** individualCount: 1; sex: female; lifeStage: adult; reproductiveCondition: mating; behavior: mating on a bark of *Populus*; occurrenceID: A0412EB2-5E33-5BCE-BE6C-36BD3DC01807; **Location:** country: Italy; municipality: Rocca de' Baldi (CN); locality: Crava-Morozzo Natural Reserve; verbatimElevation: 410 m; verbatimLatitude: 44°25'21'' N; verbatimLongitude: 7°43'26'' E; decimalLatitude: 44.4225; decimalLongitude: 7.7238888; geodeticDatum: WGS84; **Identification:** identifiedBy: Samuele Torta; **Event:** samplingProtocol: ad hoc observation; eventDate: 2024-04-25; **Record Level:** type: Stillimage

#### Distribution

The species is known with certainty for Italy only in Lazio region in a single locality where it is very abundant (Prestininzi 2009) and in Piedmont historical data during the 19^th^ century with the label "Turin" (Sommaggio 2007).

#### Notes

**Figure 7.**
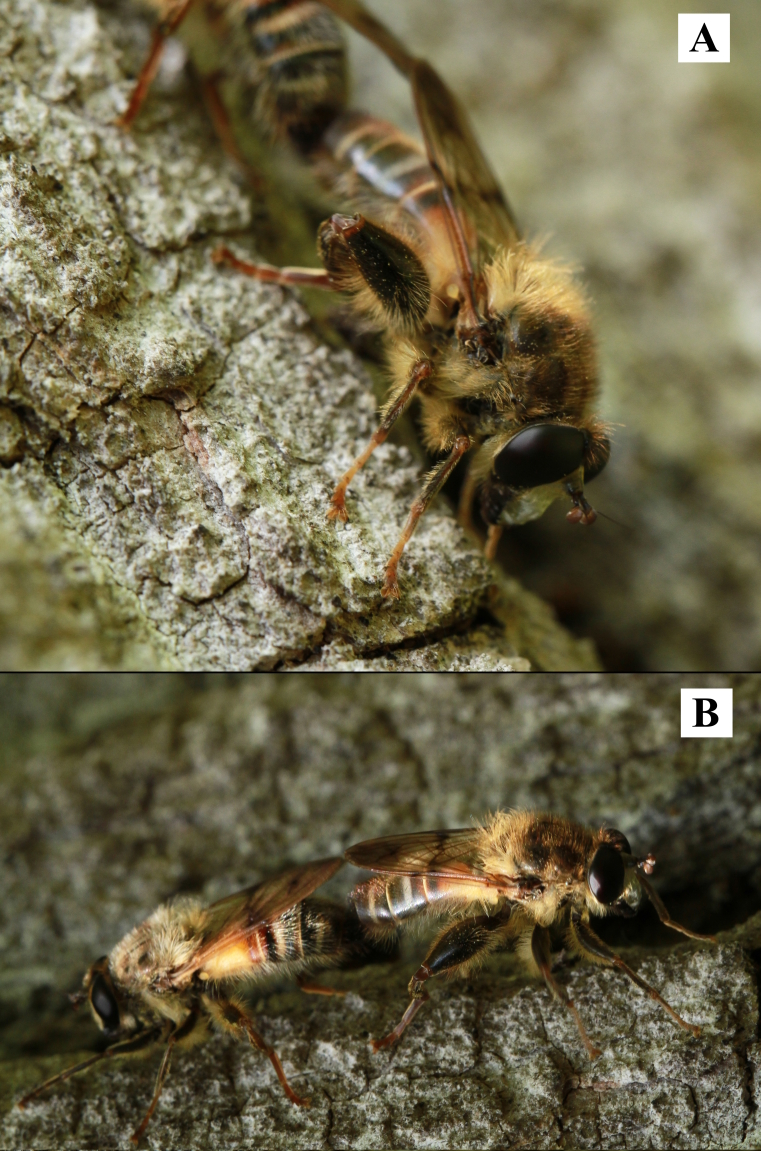
*Criorhinapachymera* on *Populus.* **A** male; **B** female on the left, male on the right.

**Figure 8.**
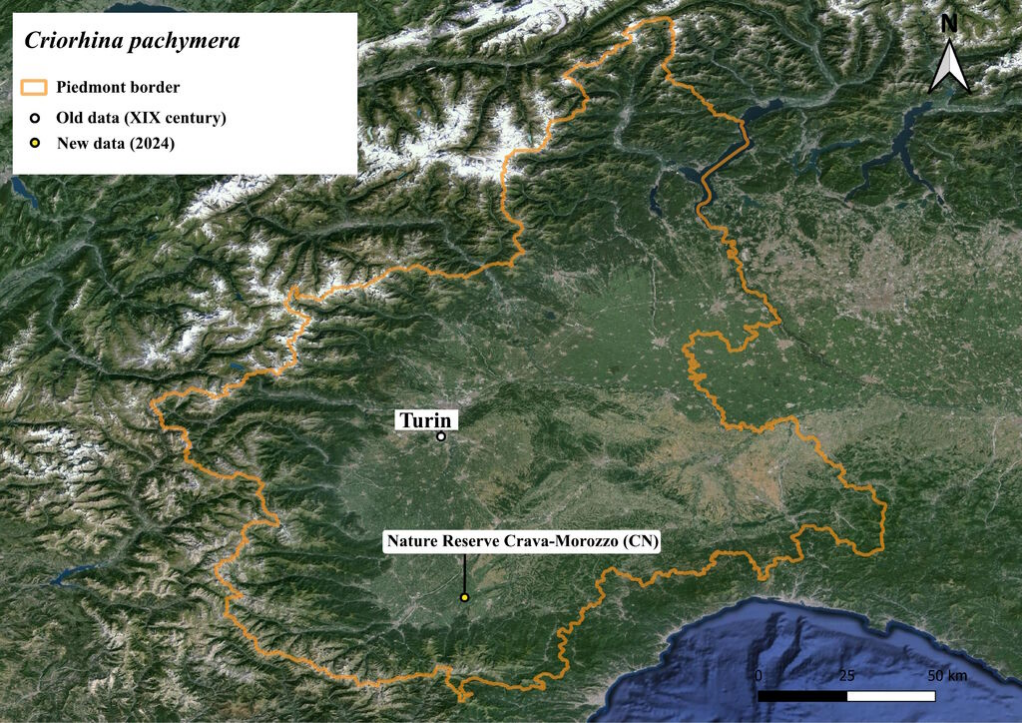
Distribution map of *Criorhinapachymera* in Piedmont region.

Six specimens of this species were collected in the Piedmont region in the 19^th^ century (Sommaggio 2007), but recent research in lowland forests failed to collect it until one of the authors (ST) took a couple of photos (Fig. 7) of a pair of specimens mating on the bark of a poplar in Crava Morozzo Natural Reserve (Fig. 8).

### Doros destillatorius

Mik, 1885

399D109E-439F-5922-BA1C-17606D4722D8

#### Materials

- **Type status:** Other material. **Occurrence:** recordedBy: Umberto Maritano; individualCount: 1; sex: female; lifeStage: adult; behavior: flying up and down near mosses; occurrenceID: BF05F808-7959-5211-BAB4-2DFC33A83E40; **Location:** country: Italy; stateProvince: Piedmont; municipality: Leinì (TO); verbatimElevation: 272 m; verbatimLatitude: 45°12'54'' N; verbatimLongitude: 7°44'01'' E; verbatimCoordinateSystem: WGS84; decimalLatitude: 45.215; decimalLongitude: 7.7336111; geodeticDatum: WGS84; **Identification:** identifiedBy: Umberto Maritan; **Event:** samplingProtocol: entomological net; eventDate: 2019-09-03; eventTime: 14:00Z; habitat: Young *Quercus* plantation; **Record Level:** type: PhysicalObject; collectionID: Umberto Maritano Collection
- **Type status:** Other material. **Occurrence:** recordedBy: Umberto Maritano; individualCount: 1; sex: female; lifeStage: adult; behavior: flying up and down on sap-run; occurrenceID: 9C4F52B0-5DC6-577A-AD08-C679822399B1; **Location:** country: Italy; stateProvince: Piedmont; municipality: Niella Tanaro (CN); locality: Valle Briaglia; verbatimElevation: 470 m; verbatimLatitude: 44°24'24'' N; verbatimLongitude: 7°53'51'' E; verbatimCoordinateSystem: WGS84; decimalLatitude: 44.4066666; decimalLongitude: 7.8975; geodeticDatum: WGS84; **Identification:** identifiedBy: Umberto Maritano; **Event:** samplingProtocol: entomological net; eventDate: 2021-09-01; eventTime: 13:00Z/14:30Z; habitat: Old *Quercus* mixed forest; eventRemarks: flying on sap-run; **Record Level:** type: PhysicalObject; collectionID: Umberto Maritano Collection
- **Type status:** Other material. **Occurrence:** recordedBy: Umberto Maritano; individualCount: 2; sex: male; lifeStage: adult; occurrenceID: 6978EE6B-92F6-5FED-97A3-08E569E844B1; **Location:** country: Italy; stateProvince: Piedmont; municipality: Valdieri (CN); locality: Casa del pescatore; verbatimElevation: 1278 m; verbatimLatitude: 44°12'56'' N; verbatimLongitude: 7°16'47'' E; verbatimCoordinateSystem: WGS84; decimalLatitude: 44.2155555; decimalLongitude: 7.2797222; geodeticDatum: WGS84; **Identification:** identifiedBy: Umberto Maritano; **Event:** samplingProtocol: Malaise trap; eventDate: 2022-09-07/13; habitat: *Fagus* forest; eventRemarks: near stream; **Record Level:** type: PhysicalObject; collectionID: Umberto Maritano Collection
- **Type status:** Other material. **Occurrence:** recordedBy: Umberto Maritano; individualCount: 1; sex: male; lifeStage: adult; behavior: foraging; occurrenceID: FAE3FF9C-CCDD-5A20-9457-AD8465A7C223; **Location:** country: Italy; stateProvince: Piedmont; municipality: Bussoleno (TO); locality: Foresto; verbatimElevation: 513 m; verbatimLatitude: 45°08'34'' N; verbatimLongitude: 7°06'52 '' E; verbatimCoordinateSystem: WGS84; decimalLatitude: 45.1427777; decimalLongitude: 7.1144444; geodeticDatum: WGS84; **Identification:** identifiedBy: Umberto Maritano; **Event:** samplingProtocol: entomological net; eventDate: 2023-09-22; eventTime: 14:50Z; habitat: scattered *Quercuspubescens*; eventRemarks: on *Hederahelix*; **Record Level:** type: PhysicalObject; collectionID: Umberto Maritano Collection
- **Type status:** Other material. **Occurrence:** recordedBy: Luciana Bartolini; individualCount: 1; sex: male; lifeStage: adult; behavior: roosting; occurrenceID: 7652D64B-3B13-5832-AD6F-63D0F8E143DC; **Location:** country: Italy; stateProvince: Tuscany; municipality: Laterina Pergine Valdarno (AR); locality: Bandella - Riserva naturale della Valle dell'Inferno e Bandella; verbatimElevation: 167 m; verbatimLatitude: 43°30'36'' N; verbatimLongitude: 11°39'30 '' E; verbatimCoordinateSystem: WGS84; decimalLatitude: 43.51; decimalLongitude: 11.6583333; geodeticDatum: WGS84; georeferenceRemarks: no exact location, maximum error 1,5 km; **Identification:** identifiedBy: Umberto Maritano; **Event:** eventDate: 2008-09-10; eventTime: 16:12Z; eventRemarks: on leaves on the ground; **Record Level:** type: Stillimage; source: https://www.lucianabartolini.net/Immagini/ditteri/sirfidi/Syrphidae-Doros.jpg

#### Distribution

**Figure 9.**
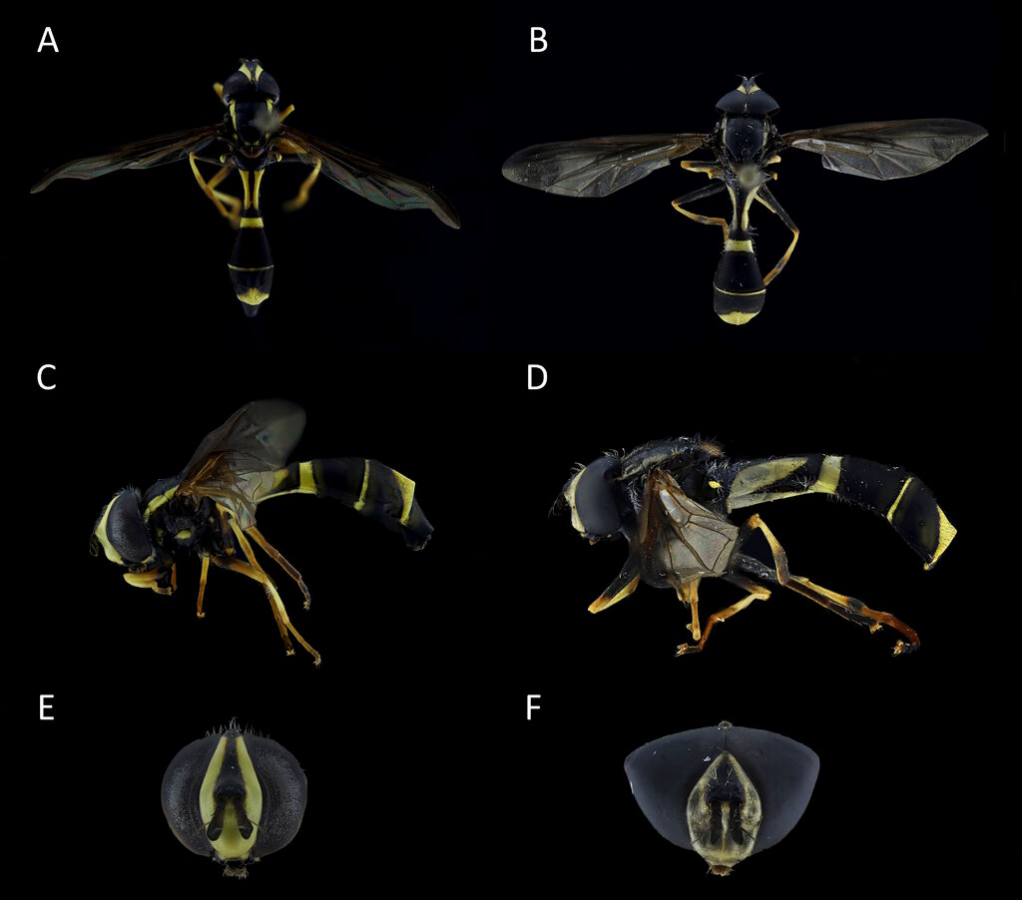
*Dorosdestillatorius* **A**, **C**, **E** female; **B**, **D**, **F** male

*Dorosdestillatorius* Fig. 9 is a rarely seen hoverfly, endangered according to the European Red List (Vujić et al. 2022), and mainly found in the Mediterranean region. It has low density populations with only 1-2 specimens collected in each known locality.

In Italy, it is known from the holotype found in Friuli-Venezia Giulia near the city of Gorizia (Speight 1987), and from the other few observations made in other regions such as: Lazio (Prestininzi 2009), Veneto (Sommaggio 2017), Valle d’Aosta, Abruzzo, Sardinia and Tuscany (Birtele 2011).

**Figure 10.**
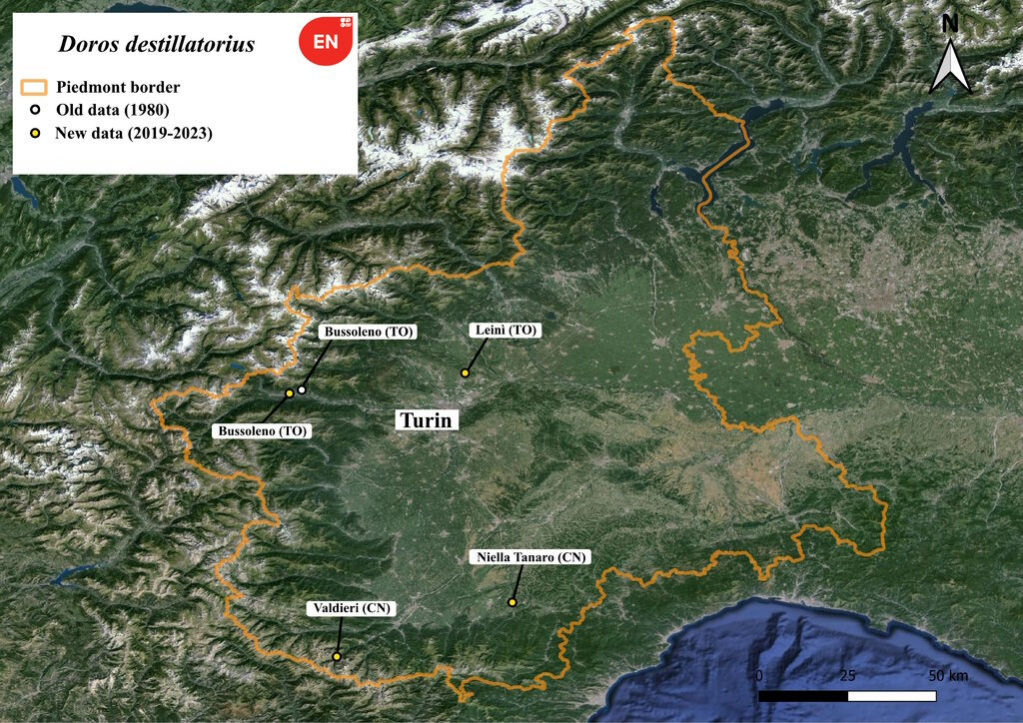
Distribution map of *Dorosdestillatorius* in Piedmont region.

From the Piedmont region, the only specimen known before this paper is from the Regional Museum of Natural Science of Turin and was collected in the year 1980 near Bussoleno (Turin Province) hamlet Pietrabianca. Fig. 10 shows all the new records of *Dorosdestillatorius* collected by the author (UM) in the Piedmont region. We report here also a new record for Tuscany.

#### Ecology

**Figure 11.**
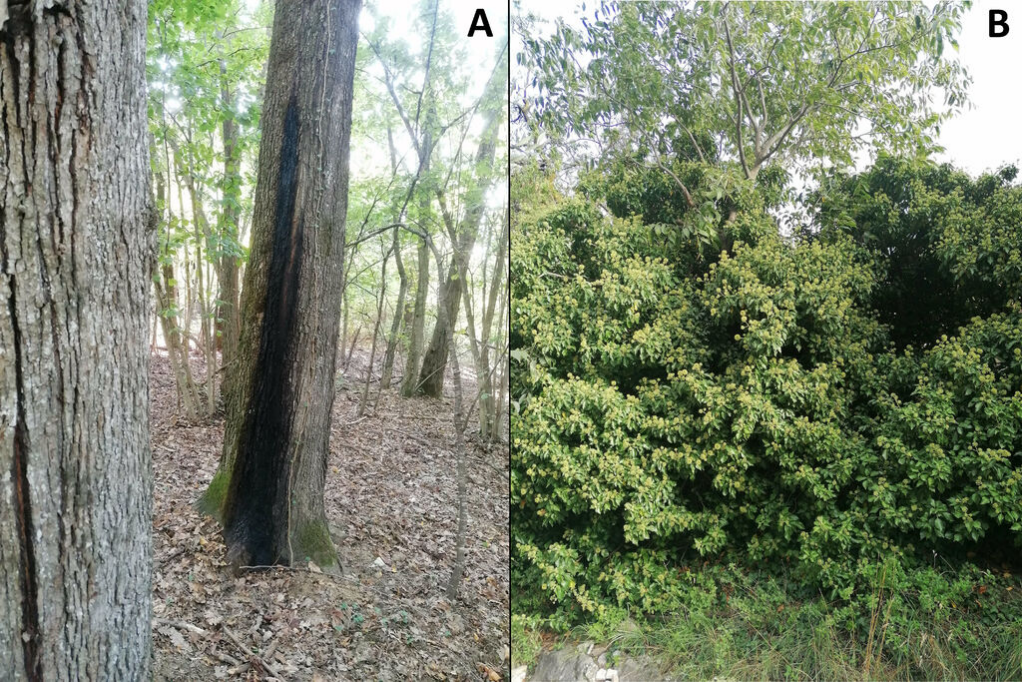
Habitats visited by *Dorosdestillatorius* . **A** Sap-run in Niella Tanaro (CN); **B** *Hederahelix* in Bussoleno (TO).

Although the surveys were conducted consistently throughout the entire season (from March to October every 10 days) in each of the reported sites, all the specimens were observed in September with females observed at the beginning of the month and males in the subsequent days. The first specimen observed (in 2019) was a female which during the hottest hours of the day moved up and down exploring mosses on the bark of a young oak tree plantation. The second specimen observed was another female (year 2021) which during the hottest hours of the day moved up and down exploring a sap-run on a large oak tree (Fig. 11). In the year 2022 a couple of males were collected by Malaise trap near a stream into a beech forest. In the year 2023, a male was seen with the aid of binoculars foraging on the top of a large ivy bloom (Fig. 11) surrounded by a landscape of few scattered *Quercuspubescens* and *Bromuserectus*. It first visited the flowers at the top of the ivy (4-5 metres above the ground) and then gradually went down to visit flowers halfway up the plant. This is the first observation of *Dorosdestillatorius* on *Hederahelix* as the only data available from literature reports it on *Rubusfruticosus* (Speight 2020).

### Hammerschmidtia ferruginea

(Fallen, 1817)

48A0BDE5-0D13-5B58-8D8E-FCE6AF2C31D2

#### Materials

- **Type status:** Other material. **Occurrence:** individualCount: 1; sex: male; lifeStage: adult; behavior: foraging; occurrenceID: C9E96EFD-5B53-55DF-B934-4E1E985B3BEA; **Location:** stateProvince: Piedmont; county: Italy; municipality: Pragelato (TO); locality: Troncea; verbatimElevation: 1756 m; verbatimLatitude: 44°57'27'' N; verbatimLongitude: 6°56'35'' E; verbatimCoordinateSystem: WGS84; decimalLatitude: 44.9575; decimalLongitude: 6.9430555; geodeticDatum: WGS84; **Identification:** identifiedBy: Umberto Maritano; **Event:** samplingProtocol: entomological net; eventDate: 2021-06-26; habitat: near Populustremula stand; eventRemarks: on *Rosacanina* flower; **Record Level:** type: PhysicalObject; collectionID: Umberto Maritano Collection
- **Type status:** Other material. **Occurrence:** individualCount: 1; sex: female; lifeStage: adult; behavior: foraging; occurrenceID: 5707B989-54B7-5E8B-B8E0-4CC78331F536; **Location:** stateProvince: Piedmont; county: Italy; municipality: Pragelato (TO); locality: Troncea; verbatimElevation: 1756 m; verbatimLatitude: 44°57'27'' N; verbatimLongitude: 6°56'35'' E; verbatimCoordinateSystem: WGS84; decimalLatitude: 44.9575; decimalLongitude: 6.9430555; geodeticDatum: WGS84; **Identification:** identifiedBy: Umberto Maritano; **Event:** samplingProtocol: entomological net; eventDate: 2021-07-17; habitat: near Populustremula stand; eventRemarks: on Apiaceae flower; **Record Level:** type: PhysicalObject; collectionID: Umberto Maritano Collection
- **Type status:** Other material. **Occurrence:** individualCount: 1; sex: male; lifeStage: adult; behavior: foraging; occurrenceID: CFA2BA4D-83AC-5521-B436-69F972E1CC0C; **Location:** stateProvince: Piedmont; county: Italy; municipality: Pragelato (TO); locality: Troncea; verbatimElevation: 1756 m; verbatimLatitude: 44°57'27'' N; verbatimLongitude: 6°56'35'' E; verbatimCoordinateSystem: WGS84; decimalLatitude: 44.9575; decimalLongitude: 6.9430555; geodeticDatum: WGS84; **Identification:** identifiedBy: Umberto Maritano; **Event:** samplingProtocol: observation; eventDate: 2021-07-17; habitat: near Populustremula stand; eventRemarks: on Apiaceae flower; **Record Level:** type: Stillimage

#### Notes

**Figure 12.**
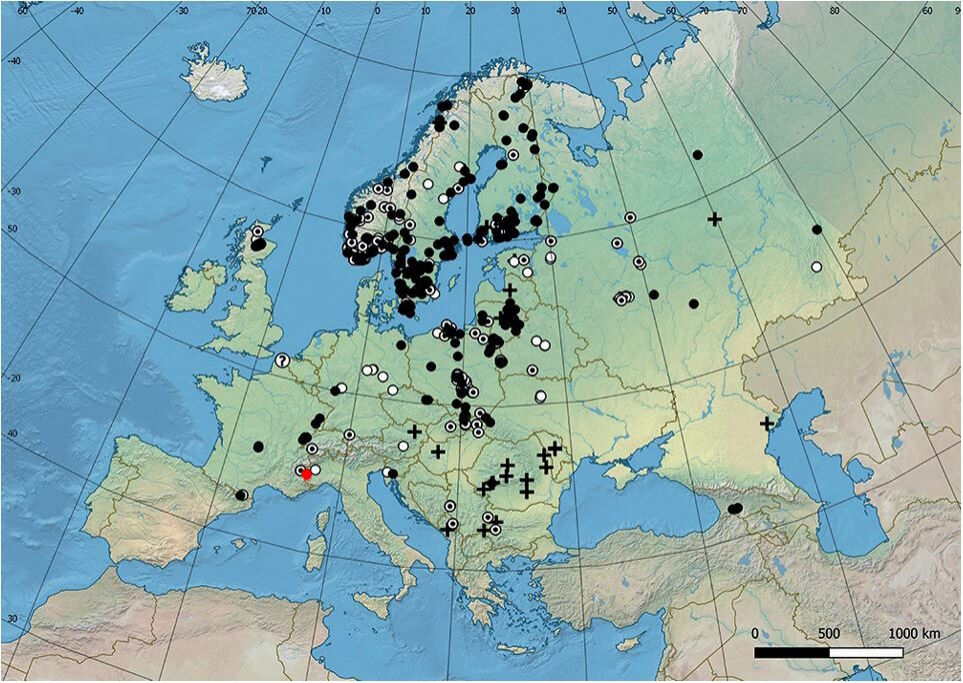
Distribution map of *Hammerschmidtiaferruginea* (white <1950, white with black point ≥1950 <2000, black ≥2000, ? = uncertain record, + datum unknown). Map modified from van Steenis (2023) with a red dot for new data.

**Figure 13.**
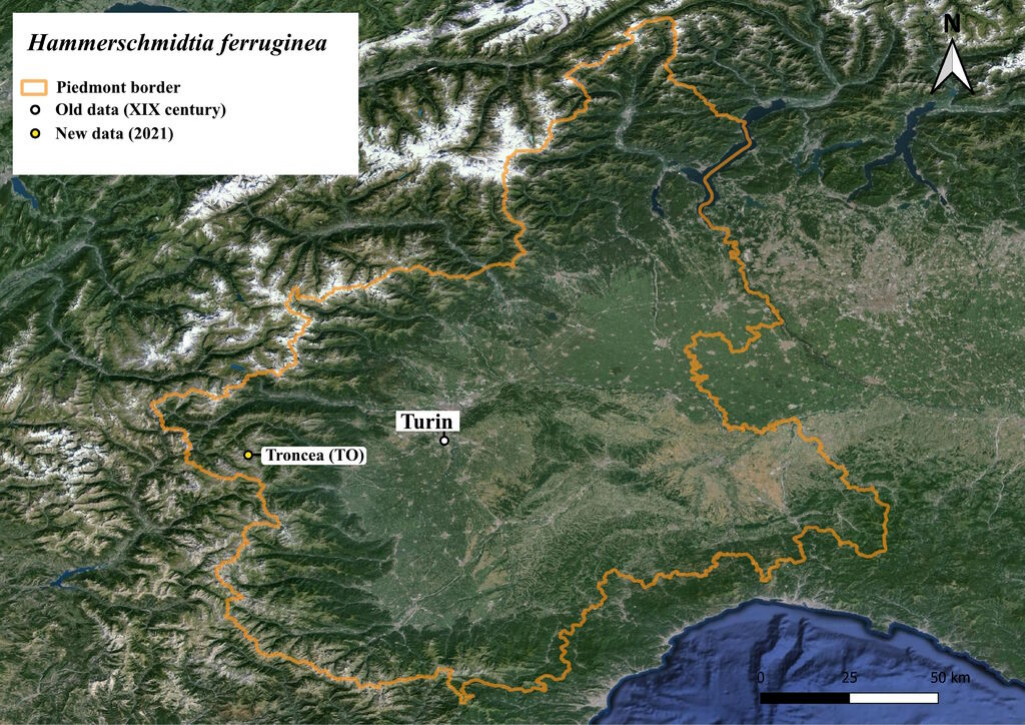
Distribution of *Hammerschmidtiaferruginea* in the Piedmont region.

**Figure 14.**
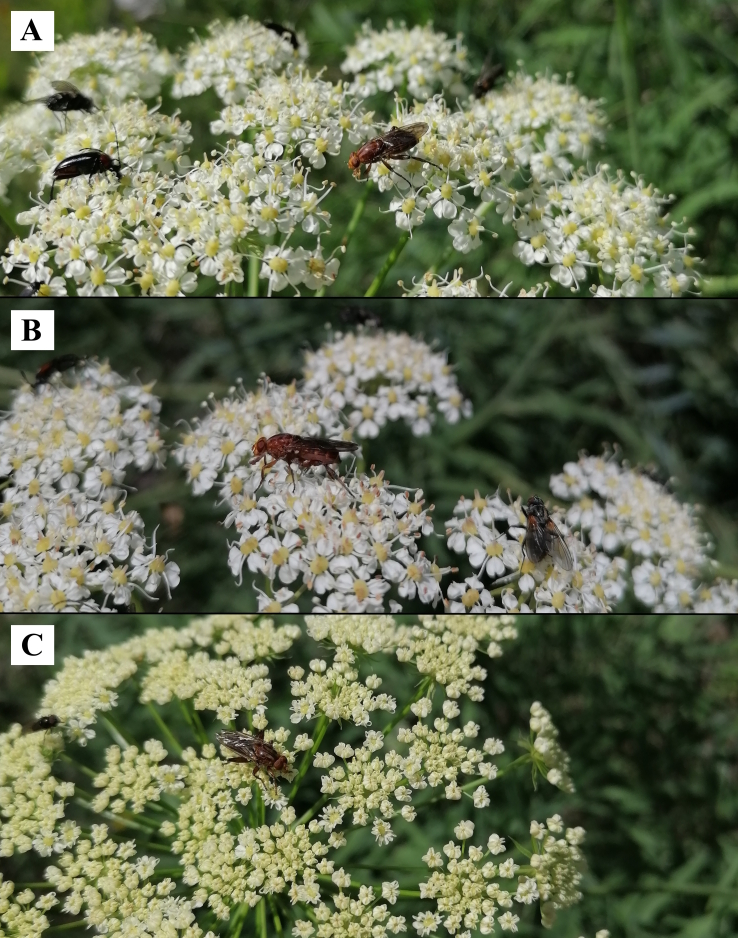
*Hammerschmidtiaferruginea* on Apiaceae flowers, 17 July 2021; **A, B** female; **C** male.

This species is widely distributed throughout the Scandinavian peninsula (Fig. 12), but it is present only in a few localities with relict populations in the Alps (Steenis et al. 2020). The only historical record for Italy dates back over a century ago near Turin (Sommaggio 2007). One of the authors (UM) found a male in June and a female and a male in July in Troncea Valley (Fig. 13). The specimens were observed in open unimproved subalpine grassland on *Rosacanina* and Apiaceae flowers (Fig. 14) foraging at a distance of 700-800 m away from a *Populustremula* stand that includes approximately 50 poplar trees in a homogeneous surface area of only 0.13 ha.

## Discussion

This study presents data on rare saproxylic hoverflies, including notable and unexpected new findings such as *Brachyopagrunewaldensis* which represents a new addition to the Italian fauna. Although *Brachypalpuschrysites* is considered mainly a high-altitude species linked to coniferous forests (Speight 2020), in Piedmont, it was previously recorded only in lowland areas undergoing reforestation where its presence is no longer confirmed. The new finding is in accordance with its trophic needs and shares the coniferous forest treeline habitat with the conspecific *B.valgus*.

*Calliceraaurata* is a widespread species in Piedmont but remains rare, with observations occurring randomly due to its high dispersal ability. Notably, sightings in Oncino and Ormea were in subalpine grassland, located at least 2 km away from any suitable larval development microhabitats. Additionally, this species proves to be polyphagous with great variability in floral preference, with colours varying from violet to white and yellow. Some observations reported in this paper are new floral preference association (Speight 2020) for the species, such as *Scabiosa*, *Fragaria* and *Carduus*/*Cirsium*.

Batesian mimicry is used by *Criorhinapachymera* (Fig. 7) to resemble the European honey bee (*Apismellifera*) subspecies. In this particular case the individuals photographed seem to match the classification made by Bisschop et al. 2023 in the B3 category, which is the brightest possible form for this species (resembling Apismelliferacf.ligustica). This is an observation that confirms the B3 as the only form known for the Italian Peninsula.

In Europe (IUCN 2021) *Dorosdestillatorius* appears to be Endangered (EN) and Critically Endangered (CR) in Czech Republic, where it is known from a single specimen collected in Podyjí National Park (Mazánek et al. 2005). A single specimen was also collected from Serbia (Vujić et al. 2018). In Italy, it is considered as Data Deficient (Burgio et al. 2015). Therefore, every newly collected data is of fundamental importance to develop and improve helpful conservation actions. The scarcity of data from all of Europe regarding this rare species emphasises the importance of considering all observations, even unpublished ones, in order to best outline the real distribution of the species and its ecological needs. Such comprehensive understanding is crucial for the conservation efforts aimed at protecting this threatened species. *Dorosdestillatorius* appears to be localised in the southern and western parts of Piedmont. Valdieri and Bussoleno sites are the only ones located within protected areas, with the latter situated near the construction zone of the Turin–Lyon High-Speed Railway megaproject. The adults were arboreal and active only for a couple of weeks at the beginning of September. They prefer both *Quercus* and *Fagus* stands, with the presence of microhabitats like mosses and sap-runs, but they are present also in woods with scattered and young trees. These differences in habitat preference make it difficult to accurately identify the ideal conditions for the survival of the species. Further research is needed to understand whether the species can be dependent on other factors such as the presence of other insects as the larvae of this species are hypothesised to be predators of aphids or myrmecophiles.

*Hammerschmidtiaferruginea* is usually found on decayed wood of *Populustremula* (the larvae microhabitat) and could be locally abundant (Rotheray et al. 2014). For the conservation of this rare species in the Alps, it would be desirable to rigorously conserve the poplar vegetation along the Troncea stream given its limited extension and status as the only known site in Italy. Increasing the presence of *Populustremula* can be achieved by establishing stepping stone patches of new plants, which would ensure the retention, maintenance and continuity of dead wood as well as the site's natural evolution.

### Conclusion

Overall, three species are reported for the first time in the Piedmont region (*Brachyopagrunewaldensis*, *Calliceraaurata* and *Calliceraspinolae*) with *Brachyopagrunewaldensis* new for the Italian fauna. Three other species of national relevance are rediscovered for the region (*Brachypalpuschrysites*, *Criorhinapachymera* and *Hammerschmidtiaferruginea*). For other two endangered species (*Callicerafagesii* and *Dorosdestillatorius*) new notable data is addressed. Knowing the distribution of threatened species is the first fundamental step in being able to act with effective conservation plans. It is of fundamental importance for threatened saproxylic hoverflies to preserve and increase microhabitat such as sup run, as in the case of *Brachyopagrunewaldensis*, and hollow trees, as in the case of *Callicera* spp. The survival of these very demanding species depends on the conservation of small microhabitats that are often point-like (van Steenis 2023, Maritano 2023).

## Supplementary Material

XML Treatment for Brachyopa grunewaldensis

XML Treatment for Brachypalpus chrysites

XML Treatment for Callicera aurata

XML Treatment for Callicera fagesii

XML Treatment for Callicera spinolae

XML Treatment for Criorhina pachymera

XML Treatment for Doros destillatorius

XML Treatment for Hammerschmidtia ferruginea
